# Complex Scenarios of Reticulation, Polyploidization, and Species Diversity within Annual Pansies of Subsect. *Bracteolatae* (*Viola* Sect. *Melanium*, Violaceae) in Italy: Insights from *5S-IGS* High-Throughput Sequencing and Plastid DNA Variation

**DOI:** 10.3390/plants11101294

**Published:** 2022-05-12

**Authors:** Anna Scoppola, Simone Cardoni, Thomas Marcussen, Marco Cosimo Simeone

**Affiliations:** 1 Department of Agricultural and Forestry Sciences (DAFNE), Tuscia University, Via S. Camillo de Lellis, 01100 Viterbo, Italy; scoppola@unitus.it (A.S.); mcsimeone@unitus.it (M.C.S.); 2 Department of Biosciences, Centre for Ecological and Evolutionary Synthesis (CEES), University of Oslo, P.O. Box 1066, NO-0316 Oslo, Norway; thmsmrcssn@gmail.com

**Keywords:** *Viola*, *Melanium*, plastid DNA, *5S-IGS* nuclear DNA, high-throughput-sequencing, evolution

## Abstract

*Viola* sect. *Melanium*, the so-called pansy, is an allopolyploid morphologically well-defined lineage of ca. 110 perennial and annual species in the northern hemisphere, characterized by markedly complex genomic configurations. Five annual pansies occur in Italy, four of which are morphologically very similar and belong to the informal ‘*V. tricolor* species complex’: *V. arvensis* (2*n* = 34), *V. hymettia* (2*n* = 16), *V. kitaibeliana* (2*n* = 16), and *V. tricolor* (2*n* = 26). Their field recognition is difficult and reflects a long-debated taxonomy often resulting in doubtful records in field inventories and across European herbaria. The current lack of comprehensive intra- and interspecific comparative studies and a relative scarcity of appropriate genetic markers coupled with unambiguous cytological descriptions are hindering clear taxa circumscription and phylogenetic inferences within this group. In this work, we tested DNA sequence variation of three highly variable plastid markers and High-Throughput Sequencing (HTS) of the nuclear ribosomal *5S-IGS* region in an attempt to decipher species identity within the *V. tricolor* species complex and to obtain an insight on their genome organization and evolution. Our results document the close relationships within this species group, a reliable molecular resolution for *V. tricolor*, and the common ancestry of *V. arvensis* and the poorly differentiated *V. kitaibeliana* and *V. hymettia*. Evidence of an important inter-population geographical divergence was recorded in *V. tricolor* and *V. arvensis*, pointing at the existence of different eco-cytotypes within these entities. Overall diversity patterns and the occurrence of two to four differently diverging *5S-IGS* lineages are discussed in the light of the acknowledged taxonomy and genomic evolutive trajectories of sect. *Melanium*.

## 1. Introduction

*Viola* L. is a large cosmopolitan genus and is considered an attractive candidate for studies of polyploid evolution [1]. The genus includes over 600 species (https://www.plantsoftheworldonline.org, accessed on 31 March 2022) distributed between two subgenera with 31 morphologically, cytologically, and geographically defined sections with ploidy levels ranging from diploid (2*x*) to at least 20-ploid (20*x*) [1]. The subg. *Neoandinium,* Marcussen et al., (ined.), is diploid (*x* = 7), but within subg. *Viola* only two out of the 20 recognized *Viola* sections have chromosome base numbers consistent with diploidy, i.e., sect. *Chamaemelanium* Ging. and sect. *Rubellium* W. Becker with *x* = 6 [1].

The subg. *Viola* is represented in the northern hemisphere by a dozen allopolyploid lineages that originated from hybridisation between two diploid lineages, referred to as CHAM and MELVIO, ca. 18 Ma ago [1]. Among these, there is the allotetraploid sect. *Melanium* Ging. No clear chromosome base number is inferable for this section, which is characterized by marked polyploidy and dysploidy based on the tetraploid level, likely indicating a considerable chromosomal remodeling during its evolution [1]. The sect. *Melanium*, the commonly called pansy, is morphologically well-defined [2,3,4] and includes ca. 110 perennial and annual north-temperate species, all but one (*V. rafinesquei* Greene in eastern North America) native to the western Palearctic [5,6,7]. Subsect. *Bracteolatae* Kupffer [8] includes four morphologically highly similar taxa, sometimes informally referred to as the ‘*V. tricolor-species complex*’ [6,7]: *V. arvensis* Murray (2*n* = 16*x* = 34), *V. hymettia* Boiss. & Heldr. (2*n* = 16), *V. kitaibeliana* Schult. (2*n* = 8*x* = 16), and *V. tricolor* L. (2*n* = 12*x* = 26) [1]. They have a long-debated taxonomy, not yet entirely resolved, e.g., [9,10,11] due to a lack of diagnostic characters, probably inflated by a marked plasticity, locally fixed variation, and a reticulate phylogenetic history due to allopolyploidy and introgression, e.g., [12,13].

*Viola tricolor* and *V. arvensis* both have European origins but are now cosmopolitan weeds. In Europe, *V. tricolor* is primarily linked to subacid sandy base-poor (but not necessarily nutrient-poor) mineral soils, whereas *V. arvensis* mainly grows on more or less nutrient-rich calcareous soils and extends to sub-acid soils at southern latitudes. *V. tricolor* is usually found in (semi)natural habitats and, at least in northern Europe, in seasonally dry grassland (subsp. *curtisii* (E. Forst.) Syme is perennial and a sand dune specialist). In south Europe, *V. tricolor* inhabits meadows (usually with subsp. *subalpina* Gaudin) and shrub fringes (with subsp. *tricolor*) [3,13,14]. *V. arvensis* occurs nearly exclusively as a weed on cultivated lands, more rarely in semi-natural habitats. Besides these two well-acknowledged species, the two closely related *V. kitaibeliana* and *V. hymettia* have been differently considered in the last two centuries. *Viola kitaibeliana* was regarded either as a subspecies/variety of *V. tricolor,* e.g., [15] (subvar. *hirta* Ging.), [16,17,18] or *V. arvensis,* e.g., [19,20,21], or as an independent species, e.g., [2,3,22,23,24,25]. The cytotype given by authors to *V. kitaibeliana s.s.* through its distribution area is 2*n* = 16 [26]. The species is widespread in central to south Europe and west Asia, with a highly fragmented distribution south- and westwards, such as in Italy and Spain [26]; A. Scoppola personal obs. Over the years, similar taxonomic treatments have been proposed for *V. hymettia*, mainly considered either a subordinate taxon within *V. tricolor*, e.g., [16] or *V. kitaibeliana,* e.g., [20] (subvar. *longeborgneana* W. Becker), [22,27], or a valid species. *Viola hymettia* is widespread in the southern Balkans and the Aegean Islands, becoming quite rare further west in central and southern Italy and Sicily [3,13]. While *V. hymettia* and *V. kitaibeliana* share the same cytotype, 2*n* = 16, recent checklists, e.g., [14,28,29] and plant databases (POWO, IPNI, The Plant List, Tropicos) treat the two taxa as independent species. In the recent treatments given for France [30,31], the “*hymettia*” type 2*n* = 16 is included in *V. kitaibeliana* as var. *trimestris* (DC.) Espeut, while previous records of *V. hymettia* Auct. for southern France, e.g., [2,7,32] are misapplied names [33], mainly attributable to a 2*n* = 48 cytotype named *V. roccabrunensis* Espeut [34].

The general poor knowledge of these four pansies results in doubtful records in field inventories and herbaria still today [13,35]. Only expert botanists can reliably identify them, based on a combination of morphological and ecological traits [3,35,36,37]. Nevertheless, additional morphs, subspecies, and presumed hybrids have been described in many local floras based on combinations of distribution range, floral, and vegetative characters. The fifth Italian annual taxon, *V. parvula* Tineo, is phylogenetically [4], cytologically, and morphologically distant from the other annuals (2*n* = 10; villous plant with inconspicuous flowers and large sepal appendages; [38]) and is placed in a different subsection (subsect. *Ebracteatae* Kupffer [8]). It was not sampled in this study.

The ‘*V. tricolor*-species complex’ is evidently a young radiation, and a relative scarcity of appropriate genetic markers is currently hindering phylogenetic inferences and clear taxa circumscription. The traditional fast-evolving nuclear ribosomal DNA (*ITS1 + 5*, *8S + ITS2*) and plastid markers (e.g., *matK*, *rpoC1*, *trnL-trnF*, etc.) have been widely used to retrieve phylogenetic relationships at the subgeneric level but with a general lack of infrasectional resolution, e.g., [36,37,39,40,41]. In addition, the use of multi-locus ribosomal DNA in polyploid and hybridogeneous taxa may be problematic due to the frequent occurrence of paralogous sequences and complex mechanisms leading to intra- and interlocus sequence evolution and homogenization [42]. Species-level molecular diversity is even harder to assess: ISSR and AFLP can provide interesting results in species delimitations [4,39], but phylogenetic inferences are made complicated by frequent hybridizations, the assumed reticulate evolution, and ploidal variability among the different species, but see [43].

Some low-copy gene regions such as *CHS* [44], *GPI* [45], *NRPD2a,* and *SDH* [1] are potential alternatives. However, their occurrence in different variants in each haplome require complex cloning procedures or multiple specific primers [46]. Thus, they can inform on the ploidy level, genome evolution, and possibly the hybrid origin of one to a few individuals, but sound taxonomic patterns based on large datasets can be difficult to obtain. In addition, issues of incomplete lineage sorting (ILS) are prominent in *Viola* and in sect. *Melanium* in particular, which along with the marked dysploidy and the taxonomic uncertainty of many taxa make the situation even more entangled. To date, no comprehensive intra- and interspecific molecular investigations have been produced on the ‘*V. tricolor*-species complex’.

Among the many available High-Throughput DNA sequencing (HTS) strategies leading to an advanced understanding of how genome variation underlies phenotypic variants [47], amplicon sequencing of loci with high phylogenetic information content [48] is a valid application to complement traditional taxonomic studies. In this view, the *5S* nuclear ribosomal RNA gene arrays (*5S-IGS* nrDNA) are a promising source of information. In plants, the hundreds to thousands *5S* repeat units per genome [49] are separated by non-transcribed intergenic spacers generally characterized by high inter-individual and intra-genomic variability. Several studies have shown that the detected variation may result in the identification of species-specific traits and complex evolutionary signals such as hybridization, introgression, and polyploidization, e.g., [50,51,52]. A couple of recent studies have generated HTS amplicon data of the nuclear *5S* spacers to improve the efficiency of signal detection and demonstrated the high potential of the technique to inspect diversity patterns and infer evolutive pathways in highly complex species systems such as Fagaceae Dumort [53,54].

In this work, we combined the DNA sequence variation data of three highly variable plastid markers and the nuclear multicopy *5S-IGS* region in a representative inter- and intra-specific sampling to provide a contribution to the taxonomy and diversification patterns of the Italian annual pansies of sect. *Melanium* subsect. *Bracteolatae* (*V. arvensis*, *V. hymettia*, *V. kitaibeliana*, and *V*. *tricolor*). Our objectives were to (1) clarify the molecular differentiation within the four species, (2) test the efficacy of the used markers for their consistent identification, and (3) obtain insights into their genome organization and evolution.

## 2. Results

### 2.1. Plastid DNA Variation

All the sequenced marker regions (*trnH-psbA*, *Rps19-trnH*, *trnD-trnY*) showed 100% unambiguous full-length electropherograms. The estimates of mean evolutionary divergence over the concatenated sequence pairs within and between taxa (Table 1) showed the lowest within group scores in *V. hymettia* and *V. kitaibeliana*; these two species also appeared the least divergent, very low diverging from *V. arvensis* but highly differentiated from *V. tricolor*.

Among the outgroup species, *V. reichenbachiana* Jord. ex Boreau (sect. *Viola* L.) was the most highly differentiated from all members of sect. *Melanium*; within this latter section, the species of the ‘*V. tricolor*-species complex’ appeared highly diverged from all other perennial species (*V. eugeniae* Parl., *V. etrusca* Erben, *V.* cf. *aetolica* Boiss. & Heldr., *V. frusinatae* Ricceri & Moraldo, and *V. aethnensis* (DC.) Strobl). An overall high number of haplotypes was obtained (37), with highest haplotype diversity in *V. arvensis* and lowest in *V. tricolor*. However, pansies of ‘*V. tricolor*-species complex’ revealed extensive haplotype sharing, especially involving *V. arvensis, V. hymettia,* and *V. kitaibeliana*. One single haplotype was shared between nine individuals of *V. tricolor* and six of *V. arvensis*.

**Figure 1:**
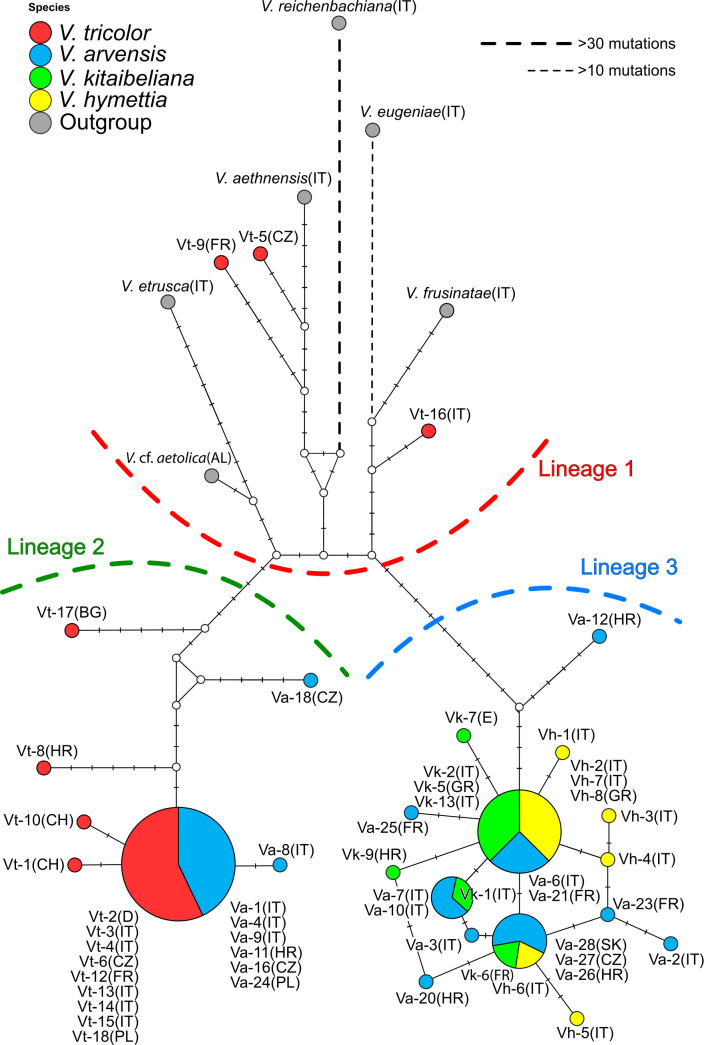
Median Joining haplotype network of the *trnH-psbA* + *rps19-trnH* + *trnD-trnY* concatenated regions in the investigated dataset. Colors identify the different species and the ecological features of each sample are reported (detailed in Supplementary File S1). Line shape and thickness indicate the relative number of mutations separating each haplotype. L1–L3 = inferred haplotype lineages.

Based on the number of mutations detected, the concatenated plastid network (Figure 1) showed three large haplotype groups separated by >10 mutations, labeled ‘Lineage 1–3’. Lineage 1 includes *V. aethnensis, V. etrusca, V. frusinate*, *V.* cf. *aetolica,* and three single *V. tricolor* haplotypes, more directly linked to the highly differentiated *V. eugeniae* and *V. reichenbachiana. Viola arvensis* haplotypes were placed in two groups: three haplotypes (8 individuals) joined the remaining five *V. tricolor* haplotypes in Lineage 2, and nine haplotypes (13 individuals) grouped with the totality of *V. hymettia* and *V. kitaibeliana* shared and single haplotypes in Lineage 3. Differently from Lineage 3, some haplotypes of *V. tricolor* and *V. arvensis*, respectively scored in Lineages 1 and 2, were separated from the nearest haplotype by >10 mutations. Based on the large extent of high intraspecific diversity and lineage mixing (in *V. tricolor* and *V. arvensis*) and the high number of shared haplotypes (in *V. arvensis, V. hymettia,* and *V. kitaibeliana*), the haplotype network appeared intricate. We tried to evaluate whether the different lineages were linked to some ecological parameters: climate, altitude, and growing habitat (Supplementary File S1). However, no clear correlation of the plastid diversity could be established. Likewise, the distribution of the three lineages has no geographical coherence.

### 2.2. 5S-IGS Nuclear Variation

Paired-end Illumina sequencing of the *5S-IGS* nuclear region in 19 *Viola* samples (14 pure and 5 samples containing pooled DNAs of 2–4 individuals) produced 825,593 total reads that were reduced to 662,632 sequences after the pre-processing steps. In order to avoid distorted information from rare unfiltered variants, all statistical analyses were performed on 421,634 sequences, corresponding to 7044 unique (representative) sequences showing an abundance ≥5 across the entire dataset (Supplementary File S2). No *Viola 5S* rDNA or intergenic spacer sequences are currently available in gene banks such as the NCBI nucleotide archive (https://www.ncbi.nlm.nih.gov/; accessed on 15 December 2021). The obtained dataset revealed 95–100% identity of the first 40–60 and last 30–50 sequenced bp with the 3′ and 5′ ends of several diverse plant nuclear-encoded *5S* rRNA gene (*5S* rDNA) sequences, including *Brassica* L., *Chenopodium* L., *Oenothera* L., *Vitis* L., *Jasminum* L., and *Rubus* L. The overall mean genetic distance of the entire HTS dataset in these two sequence portions calculated as the number of base differences per site by averaging over all sequence pairs was very low (0.01). We therefore concluded that the two regions belong to the 3′ and the 5′ portions of the highly conserved *5S* rRNA gene. In contrast, no high similarity BLAST scores were detected for the *IGS* region comprised between these two portions. Sample occurrence and main structural features of the used sequences (length and GC content) are detailed in Supplementary File S2.

Table 2 and Table 3 report the mean evolutionary divergence estimated for the samples containing DNA from single individuals. The intra-genome variation in *V. arvensis*, *V. kitaibeliana,* and *V. hymettia* is very low and similar to their intra- and interspecific mean divergence. In contrast, the *V. tricolor* samples have higher values of intra-genomic and intra-specific distance and appear well differentiated from the *V. arvensis-kitaibeliana-hymettia* species set.

The same analysis was performed on all samples of the ‘*V. tricolor*-species complex’, including samples with multiple individuals pooled according to their geographical origin (Table 4). The results highlighted the divergence of all the *V. tricolor* samples across the entire dataset and revealed the occurrence of interesting intra-specific differentiation in the other species, e.g., the higher divergence scored by samples T10 (*V. arvensis* from Italy), T28 (*V. hymettia* from Greece), T20, and T21 (*V. kitaibeliana* from Greece and Spain) compared to the other conspecific samples. In general, the Italian samples of *V. kitaibeliana* and *V. hymettia* were all poorly differentiated and especially samples T24–T27 (*V. hymettia*) and T17 (*V. kitaibeliana*) shared a very low genetic divergence. Compared with their conspecifics, the two sympatric samples of both species (T20 and T28 from Greece) displayed higher divergence estimates.

A large part of the *5S-IGS* sequences was shared among multiple species and samples (Supplementary File S2). Based on their relative occurrence across samples, we defined four ‘specific’, four ‘local’, and four ‘ambiguous’ classes. ‘Specific’ sequences are 99.95–100% exclusive of a single species and only shared among conspecific samples; ‘local’ sequences were exclusively found in single samples and could not be unambiguously assigned to any species. The ‘ambiguous’ classes were defined as: (1) ‘Sect *Melanium’*, shared between at least one outgroup and one individual belonging to the ‘*V. tricolor*-species complex’; (2) ‘Gr. Tricolor’, shared between *V. tricolor* and at least one individual of the *V. arvensis-hymettia-kitaibeliana* species’ set; (3) ‘Gr. Arvensis’, shared between *V. arvensis* and *V. hymettia* or *V. kitaibeliana*; (4) ‘Kitaibeliana/Hymettia’, shared only among these two species. Three additional ‘specific’ classes were assigned to the outgroups (*V.* cf. *aetolica*, *V. eugeniae*, *V. frusinatae*).

**Figure 2:**
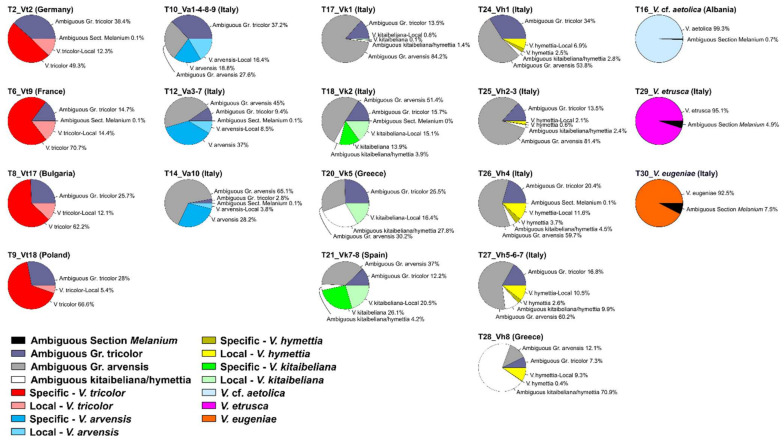
Proportional occurrence of the different *5S-IGS* sequence classes (sequence abundance ≥5) in the investigated samples.

We then quantified these sequence classes in each investigated sample and maintained the same classification in the following phylogenetic analyses. The distribution of the specific and ambiguous *5S-IGS* sequences in the 19 samples is shown in Figure 2. ‘Ambiguous Sect. *Melanium*’ sequences are nearly absent in the ‘*V. tricolor*-species complex’. In contrast, all the samples of this species’ group share large proportions of the remaining ambiguous *5S-IGS* variants. The ‘Gr. Tricolor’ sequences occur in each sample of the ‘*V. tricolor*-species complex’, with highest and lowest occurrence in two samples of *V. arvensis* (T10 = 37.2%, T14 = 2.8%, respectively). Likewise, the ambiguous ‘Gr. Arvensis’ sequences occur in each sample of the three involved species, totalling 27.6–65% in *V. arvensis*, 30.2–84.2% in *V. kitaibeliana,* and 12.1–81.4% in *V. hymettia*.

These two latter species also share 1.4–70.9% of sequences. Species-specific sequences were detected in variable amounts in the different species, with highest proportions in *V. tricolor* (49.3–70.7% + 5.4–14.4% ‘local’ sequences), followed by *V. arvensis* (18.8–37% + 3.8–16.4% ‘local’ sequences). *Viola kitaibeliana* (with 0–26.1% ‘specific’ and 0.8–20.5% ‘local’ sequences) and *V. hymettia* (with 0.4–3.7% ‘specific’ and 2.1–11.6% ‘local’ sequences) scored the lowest levels of local and specific sequences and evidenced some variation. For instance, one Italian and the Iberian samples of *V. kitaibeliana* (T18, T21) revealed relatively high proportions of ‘specific’ (13.9–26.1%) and ‘local’ sequences (15.1–20.5%). In sharp contrast, the other Italian and the Greek samples showed, respectively, negligible amounts of ‘specific’ and ‘local’ sequences with higher amounts of ‘Ambiguous Gr. arvensis’, and no ‘specific’ but very high amounts of ‘Ambiguous Kitaibeliana-Hymettia’ sequences. The same situation was detected in *V. hymettia*, with some samples showing a homogeneous class composition (e.g., samples T26 and T27), and others with an excess of ‘Ambiguous Gr. Tricolor’ (T24), ‘Ambiguous Gr. Arvensis’ (T25), and ‘Ambiguous Kitaibeliana/Hymettia’ (T28) sequences.

### 2.3. Nuclear Phylogenetic Analysis

For the subsequent phylogenetic analyses, we combined all obtained sequences with abundance ≥25, removed 88 outlier variants shorter than 280 bp (see Supplementary File S2) and three additional sequences that were poorly alignable to the rest (ca. 8% of the obtained dataset).

**Figure 3:**
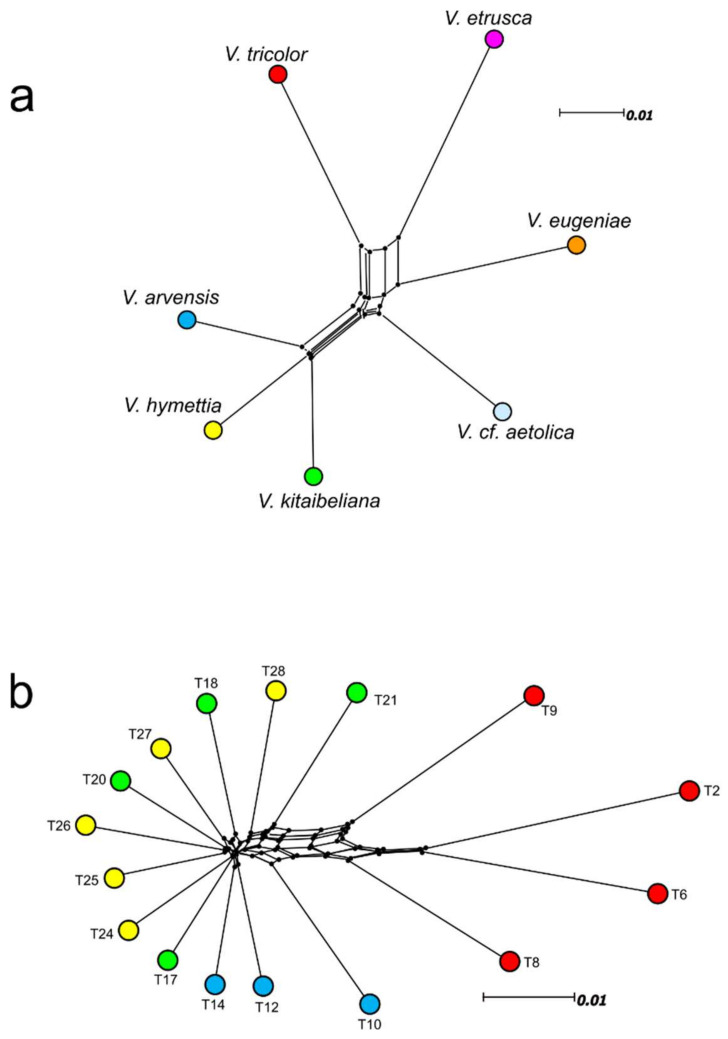
Neighbor-Net splits graph generated with the *5S-IGS* sequences filtered for abundance (≥25) and length (≥280 bp) in all investigated species (**a**), and all samples belonging to the ‘*V. tricolor* species complex’ (**b**).

The N-Net splits graphs between species and samples are reported in Figure 3a,b, and show a pronounced trunk separating *V. arvensis*, *V. kitaibeliana,* and *V. hymettia* from *V. tricolor* and the perennial pansies. The overall pattern in the splits network is quite tree-like except for a conspicuous split that places the high-polyploid *V. etrusca* (2*n* = 40) intermediate between *V. tricolor* and *V. eugeniae*. The sample-wise network (Figure 3b) highlighted more conflicting signals between the investigated populations, but confirmed the separation of *V. arvensis*, *V. kitaibeliana,* and *V. hymettia* from *V. tricolor*.

Nevertheless, separation of the *V. arvensis* samples was slight and all *V. kitaibeliana*-*hymettia* samples were highly intermingled, thus confirming the results obtained with the total *5S-IGS* sequence dataset (Table 4).

**Figure 4:**
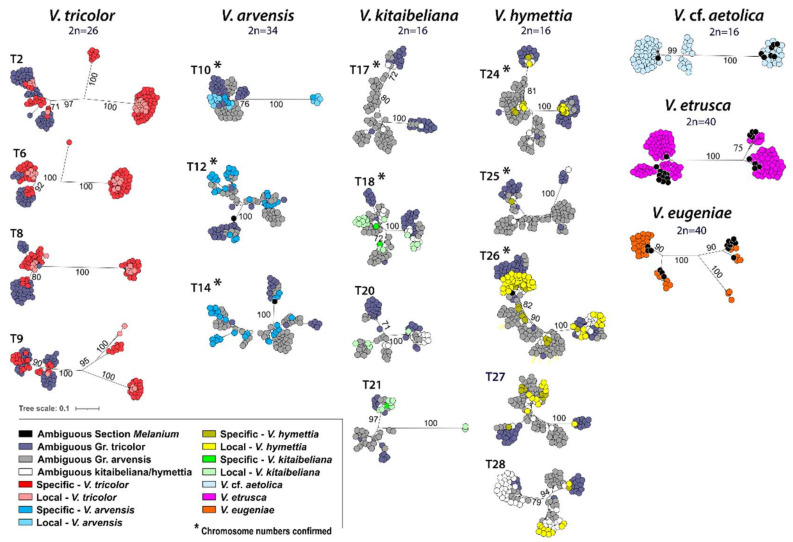
Unrooted maximum likelihood phylograms of the *5S-IGS* sequences, filtered for abundance (≥25) and length (≥280 bp), in each sample; sequence class composition and the chromosome number of species/samples are reported.

The unrooted *5S-IGS* RAxML trees of the 19 samples (989 tip-set) are shown in Figure 4. The generated 5S-HTS sequences grouped in a variable number of high-supported (70–100% BS) major clusters in each sample. The number of the clusters identifies different intra-individual lineages and could be related with the number of 5S loci occurring in each sample [54]. In agreement, the four samples of *V. tricolor* displayed a variable number of major clusters (three clusters in sample T8; three clusters and a single high-diverging variant in sample T6; four clusters in samples T2 and T9), perfectly matching the ‘six to eight 5S rDNA signals on six chromosomes’ detected in different *V. tricolor* populations across Europe with FISH [55]. Unfortunately, the current lack of FISH data, coupled with the pronounced intra-taxon heterogeneity observed in this study, prevents any inference on the 5S loci number in the other members of the ‘*V. tricolor* species complex’. In *V. arvensis*, three major clusters were scored in sample T10 (BS = 76–100%) and two major clusters (one of which largely subdivided in multiple low-divergent sub-clusters) in samples T14 and T12. Likewise, a high number of low-divergent subclusters complicated the identification of major lineages in *V. kitaibeliana* (possibly amounting to three, in samples T18, T20, T21, and four, in sample T17) and *V. hymettia* (two to four major clusters with additional subclusters). In contrast, three and four major clusters were clearly scored by the *V. etrusca*, *V.* cf. *aetolica*, and *V. eugeniae* samples, respectively.

**Figure 5:**
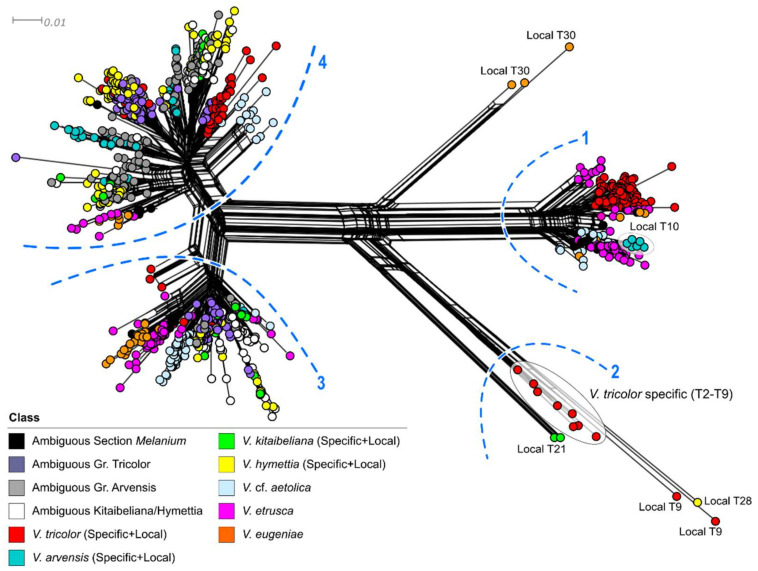
Neighbor-Net splits graph of the 989 *5S-IGS* sequences filtered for abundance (≥25) and length (≥280 bp) in the investigated dataset. Identified sequence clusters are numbered 1 to 4.

The Neighbor-Net graph (Figure 5) confirms the above data and clarifies the phylogenetic relationships of the obtained *5S-IGS* dataset. All the obtained sequences group in four major groups, labeled Cluster 1 to 4, and an additional minor one identified by only three *V. eugeniae* sequences (unlabeled). Specific sequences of *V. eugeniae* and *V. tricolor* are placed on four clusters, one of which (Cluster 2) collected ‘specific’ *V. tricolor* sequences shared only by samples T2 and T9. Specific *V. etrusca* and *V.* cf. *aetolica* sequences grouped on Clusters 1, 3, and 4 and the *V. hymettia* and *V. kitaibeliana* specific sequences largely identified Clusters 3 and 4 (each one subdivided in 2–3 subclusters), with only two local sequences of *V. kitaibeliana* (sample T21, Spain) and one of *V. hymettia* (sample T28, Greece) sharing more affinity with Cluster 2. Specific *V. arvensis* sequences are also placed on three clusters (1, 3, 4), but Cluster 1, comprising only sequences belonging to *V. tricolor* and the three outgroup species, is only targeted by ‘local’ sequences of sample T10. ‘Ambiguous Sect. Melanium’ sequences are placed on Clusters 1, 3, and 4, whereas ambiguous ‘Gr. Tricolor’, ‘Gr. Arvensis’, and ‘Kitaibeliana/Hymettia’ sequences grouped only on Clusters 3 and 4. We may therefore derive the following numbers of major *5S-IGS* sequence lineages in the investigated dataset: four in *V. eugeniae*, three and four in *V. tricolor*, three in *V. etrusca* and *V.* cf. *aetolica*, two and three in *V. arvensis*, two in *V. hymettia* and *V. kitaibeliana*.

**Figure 6:**
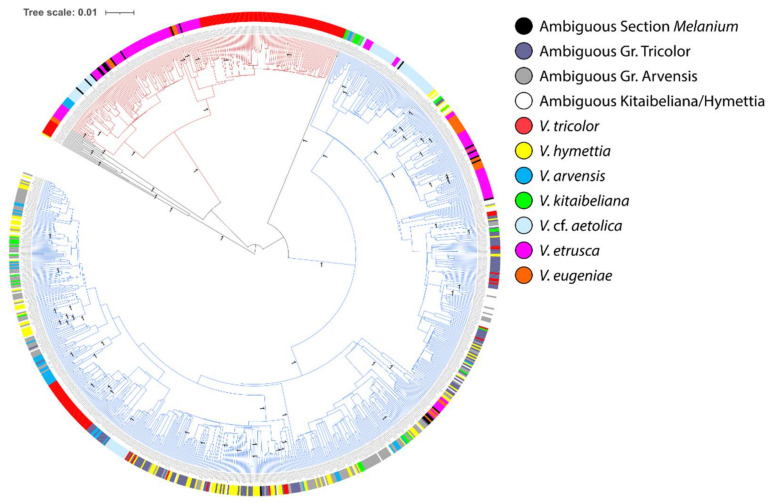
BEAST phylogenetic dendrogram of the 989 *5S-IGS* filtered for abundance (≥25) and length (≥280 bp) in the investigated dataset. Nodes Posterior Probabilities are reported. Major deep clades are evidenced in red and blue.

Finally, the BEAST analysis (Figure 6) revealed two deep clades. Sequences of all species are highly mingled and the three highly diverging *V. kitaibeliana* (‘Local T21’) and *V. hymettia* (‘Local T28’) sequences are at the root of the two clades, each one further subdivided in two major and several minor subclades. The first deep clade (red-colored in Figure 6) included sequences of sample T10 (*V. arvensis*) and all other species with the exception of *V. kitaibeliana* and *V. hymettia*. Specific and shared sequences of these two species with *V. tricolor* and *V. arvensis* (Ambiguous ‘Gr. Tricolor’ and ‘Gr. Arvensis) were exclusively accommodated in multiple subclades of the second deep clade (blue-colored).

## 3. Discussion

### 3.1. Plastid DNA Differentiation

Many studies have been carried out to address phylogenetic and taxonomic questions at the infrageneric level in Viola, e.g., [1,40,43,56]. Our study is the first to report data from extensive intra- and interspecific investigations on a specific group within sect. *Melanium* in a well-defined regional scale (i.e., the Italian pansies of subsect. *Bracteolatae*). It is now well acknowledged that the chloroplast genome is conservative in land plants, and its main utility may be not just to achieve taxonomic identification but rather to retrieve phylogeographic patterns that can be used to infer the evolution of related lineages and hybridization/introgression events [57]. In this work, we detected a large variation at the targeted plastid loci, confirming the high variation data from the comparative chloroplast genome analysis in five East Asian Viola species [56]. However, despite the high variability generally observed (Table 1), sequence diversity differentiating *V. arvensis*, *V. kitaibeliana*, and *V. hymettia* is low. In addition, the large extent of haplotype sharing between *V. tricolor* and *V. arvensis* and among *V. arvensis*, *V. kitaibeliana*, and *V. hymettia* prevents any molecular circumscription of the species identity. Likewise, the geographic distribution of the haplotypes does not allow the recognition of clear geographic or ecological patterns (Figure 1; Supplementary File S1). In general, all gathered plastid data point towards the likely retention of ancestral variation within the ‘*V. tricolor* species complex’ (especially in *V. tricolor*/*V. arvensis* and *V. arvensis*/*V. kitaibeliana*/*V. hymettia*) as a result of incomplete lineage sorting, polytopic allopolyploid origins, or past interspecific gene flow within this group of largely sympatric pansies.

Geographically close populations of all species generally fall in different haplotype groups, whereas distant populations share the same haplotypes, supporting the idea of different locally adapted intra-specific lineages. In agreement, *V. tricolor* s.l. is known to occur in Europe with different morphological (e.g., var. macedonica (Boiss. & Heldr.) P. D. Sell, subsp. *tricolor* var. polychroma (A. Kerner) Gams), ecological (inhabiting sandy dunes, meadows, fringes, clearings and wood edges, fields, heavy metal soils, e.g., subsp. subalpina, var. *raiblensis* D. Lausi & T. Cusma Velari, subsp. curtisii), phenological (with annual, short-lived biennial, or perennial habit) and cytological forms, natural hybrids, and introgressed individuals [12,36,55,58]. Besides some acknowledged *V. tricolor* subsp. tricolor and *V. tricolor* subsp. subalpina samples ([38] (Supplementary File S1), some of these variants, especially from outside Italy, could be indeed an unnoticed part of our dataset. Likewise, the two haplotype groups found in *V. arvensis* (one more closely related to V. tricolor, the other to *V. hymettia* and *V. kitaibeliana*) may highlight the occurrence of two different gene pools underlying different forms. The field pansies in the two haplotype groups effectively grow in different habitats (Supplementary File S1) and possess different nuclear genetic composition (see below) which would further support the identification of a new entity in Italy, in addition to the typical *V. arvensis*, matching previous morphological inferences by Scoppola et al. [59].

We may therefore assume that a combined effect of common ancestry, hybridization, unhindered post-glacial migration (of the more widely distributed haplotypes), isolation, and adaptation (of rare haplotypes) with different responses to an increasing anthropic influence (land use change, livestock farming, etc.,) can be a possible explanation for the observed complex patterns. There are several considerations in support of a complex interplay of these multiple evolutionary and bio-ecological factors. Reticulation and a high phylogenetic affinity of the entire *V. tricolor*-species group has been widely inferred [1,4]. Past and recent hybridization as in other Viola sections [60,61] is possible, coupled with a differential efficiency of species-specific reproductive strategies (most pansies are allogamous, highly adapted to entomophilous pollination, other species including *V. arvensis* and *V. kitaibeliana* are reported as predominantly autogamous [4,62,63]). Persistence in multiple glacial refugia across Europe has been postulated [64], and colonization of synanthropic habitats, with local adaptation possibly favoured by a limited (diplochorous) seed dispersal capability has been observed [35].

More extensive future sampling may expand our comprehension of the tangled plastid phylogeography in sect. *Melanium*. However, field sampling across large territories can be extremely complex in pansies and the large amount of specimens deposited in herbaria can be poorly informative or not correctly assigned. In this work, for instance, we revised some specimens referred by authors/collectors to *V. kitaibeliana* and assigned them to *V. arvensis* (i.e., Va25, Va26, Va27, and Va28) and a specimen referred to *V. arvensis* from Albania was assigned to *V.* cf. *aetolica*. Precise cytotype assessment and accurate specimen identification would be an ideal preliminary asset (see for instance [65]). On the other hand, although our outgroup sampling was extremely limited, the selected plastid markers appear promising for differentiating species of different sections and, possibly, the perennial pansies.

### 3.2. Nuclear 5S-IGS Polymorphism

The short non-transcribed intergenic spacer of the 5S nuclear ribosomal RNA gene array is the most-divergent nuclear marker known today, e.g., [50,66,67,68,69,70]. More interestingly, due to its extreme inter-individual and intra-genomic variability, the multi-copy, potentially multi-locus, 5S rDNA has the potential to store signals from reticulate evolutionary processes and intra-genomic recombination, thus allowing the detection of diversity patterns and evolutionary inferences of complex species systems, e.g., [52,53,54,71].

In this work, we performed HTS sequencing of the 5S amplicon in single (for a clearer view of intra-individual variation) and multiple (to expand the inter-individual investigation) Viola conspecific samples. No data from other Viola or Violaceae taxa was available for a useful structural (and phylogenetic) comparison. Likewise, little is known about the GC content in non-coding intergenic spacers of functional 5S repeat units [72]; we therefore filtered our sequences based on their abundance and length, in addition to the standard pre-processing procedures, to avoid rare pseudogenic variants or artefacts that could have produced distorted results in our phylogenetic analyses. The sequence and length variability of the *5S-IGS* HTS data used for the phylogenetic analyses is in agreement with all known evidence in many different plant groups, e.g., [50,51,66,68,69,73,74,75]. The estimated intra-genome diversity in the four species of the ‘*V. tricolor*-species complex’ (Table 2) increases with the chromosome number of the samples and corresponds to similar scores of intra-specific mean genetic diversity, indicating a substantial genome homogeneity across conspecific samples and the occurrence of different *5S-IGS* lineages in the various specific haplomes. However, our results document an unexpectedly tangled genetic diversity, with *V. arvensis*, *V. kitaibeliana*, and *V. hymettia* appearing genetically very similar and equally high-divergent from *V. tricolor* (Table 3; Figure 3) and with a large portion of the *5S-IGS* variants shared at various degrees at the interspecific level (Supplementary File S1). All these findings point to a common ancestry of these three taxa with respect to the other investigated species, possibly exacerbated by low diversity and past and reiterated allopolyploidizations [1,4]. Our sequence categorization in interspecifically shared, sample-unique, and species-specific classes, and their distribution in the different samples (Figure 2), showed that the ‘Ambiguous Sect. Melanium’ variants are absent in *V. kitaibeliana* and *V. hymettia* and only present in negligible amounts in few samples of *V. tricolor* and *V. arvensis* (samples T2, T6 and T12, T14, respectively), thus indicating a nearly full differentiation of this species set from the perennial pansies. In contrast, all ‘Ambiguous Gr. Tricolor’ sequences, largely shared among the ‘*V. tricolor*-species complex’, witness a common genetic background, likely representing the heritage of a linked origin, as well as the ‘Ambiguous Gr. Arvensis’ sequences, exclusively shared among *V. arvensis*, *V. kitaibeliana,* and *V. hymettia*. The four taxa of the ‘*V. tricolor*-species complex’ therefore confirm to constitute a closely linked group and the *V. arvensis*-*hymettia*-*kitaibeliana* species set represents a further subgroup. Local hybridization or introgression phenomena cannot be discarded, although the extent of sequence sharing (ambiguous ‘Gr. Arvensis’ and ‘Hymettia-Kitaibeliana’) seems to be too widespread and not related to geographic proximity. Interestingly, the highest proportions of ‘Ambiguous Kitaibeliana/Hymettia’ sequences were shown by the two Greek samples of both species, one of which (T28, *V. hymettia*) was collected at the species’ locus classicus (legit E. Baliousis 2019, UTV). However, the two samples are genetically different (Table 4, Figure 3b), indicating that the ‘Ambiguous Kitaibeliana/Hymettia’ sequences are mainly shared with the remaining conspecific samples. Unfortunately, no sympatric *V. arvensis* samples were available for a comparison that could have eventually helped re-assign this ambiguous sequence class to ‘Gr. Arvensis’, similarly to the Italian samples. In contrast, both *V. kitaibeliana* and *V. hymettia* showed a homogeneous *5S-IGS* sequence class composition across all Italian samples (Figure 2), low amounts of specific sequences, and very low intra- and inter-specific genetic distances among them and with *V. arvensis* (Table 4), indicating an overall poor differentiation of these two species on the Italian territory (Figure 3b). Higher amounts of species-specific sequences were found in *V. tricolor* and *V. arvensis*, with sample T10 differing from the conspecifics in terms of class composition (see the high proportion of ‘Ambiguous Gr. Tricolor’ sequences) and mean divergence from the other *V. arvensis* samples. Sample-exclusive (‘local’) sequences occurred in different proportions across all samples; they exceeded the species-specific sequences in (nearly) all *V. kitaibeliana* and *V. hymettia* samples, in sharp contrast with *V. tricolor*, *V. arvensis* and the (assumed; see below) *V. kitaibeliana* individual from Spain (T21). Such a differential occurrence of ‘specific’ (shared among conspecific samples), ‘ambiguous’ (shared among species), and ‘local’ (sample exclusive) sequences can indeed be related with high rates of inter-population gene flow among genetically rich species (*V. tricolor* and *V. arvensis*) and isolation among genetically poorer species (*V. kitaibeliana* and *V. hymettia*). Introgression among these latter species is obviously possible, as well as among sample T10 and *V. tricolor*, not excluding sample misidentification and/or a possible misuse of a uniform taxonomy across the collectors or herbaria that provided the specimens. Even if increased intra- and inter-specific sampling might reveal larger sequence sharing and allow a redefinition of the ‘local’ into ‘specific’ sequence classes, and/or expand the proportion of ambiguous sequences, all gathered results confirm the common origin of the ‘*V. tricolor* species complex’ and point towards a molecular circumscription of the *V. arvensis*, *V. kitaibeliana,* and *V. hymettia* species group. Consistent variation at the population/geographic level is evident; however, the two latter species appear genetically depleted and difficult to resolve (Table 2, Table 3 and Table 4; Figure 3a,b).

### 3.3. Inferences on Annual Melanium Taxonomy

The least divergent samples of *V. kitaibeliana* and *V. hymettia* from *V. arvensis* are all Italian; the higher divergence estimates of the Greek (T20, T28) and Spanish (T21) samples, and their different *5S-IGS* variant composition, may be therefore explained with geographical distance. Sample T21, in particular, reveals the highest amount of ‘specific’ sequences (i.e., shared among different *V. kitaibeliana* samples). It is worth noting that the T21 individual comes from a population with large and intensely colored corollas, whose attribution to *V. kitaibeliana* is not entirely certain (J. López Tirado, personal communication). At the same time, all the Italian *V. hymettia* samples appeared rather uniform (low genetic divergence and similar *5S-IGS* sequence composition) and the Italian samples of *V. kitaibeliana* (T17, T18) are very low divergent from them, moderately divergent themselves and from *V. arvensis* (especially sample T17), and include different amounts of species-specific *5S-IGS* variants, with the lowest proportion in sample T17. Therefore, *V. hymettia* and (at least) some populations of *V. kitaibeliana* seem to constitute, in Italy, a unique entity, closely linked to *V. arvensis*, from which they would however differ in chromosome numbers and morphology. We may therefore question whether the Italian populations of *V. hymettia* may have been erroneously attributed to the Greek taxon, although sharing the same cytotype and similar morphology. In agreement, these populations have been described as ‘atypical’ [38], and it has been suggested that all central-west, southern European pansies with large corollas and cytotype 2*n* = 16, as recorded in France, should be rather attributed to *V. kitaibeliana* var. *trimestris* (DC.) Espeut [30,31]. Indeed, the large geographic distances among sampling sites, and their location in three different glacial refugial macro-areas (Iberian, Italian, and Balkan peninsulas), might explain the genetic (and morphological) diversity detected among and within the *V. hymettia* and *V. kitaibeliana* samples. We may also speculate that drifting, adaptation to different habitats, and isolation may have caused some phenotypic diversity, witnessed by the various amounts of ‘specific’ and ‘local’ sequences. Their most correct taxonomic rank is therefore yet to be assessed. Broader sampling of all pansies with large corollas and cytotype 2*n* = 16 that occur within the vast distribution range of *V. kitaibeliana*, and especially focused on the Euro-Mediterranean area where *V. hymettia* preferentially grows, would clarify these issues and further define the real identity of ‘Ambiguous Kitaibeliana/Hymettia’ and ‘local’ sequence classes to definitely assess the identity of the Italian entities.

An important finding of our work resides in the two detected genetic clusters within *V. arvensis*. The two genetic variants differ both at the plastid (two highly different lineages; Figure 1) and nuclear level (different composition and high divergence of the *5S-IGS* sequences compared with the other conspecific samples; Figure 2 and Figure 4, Figure 5 and Figure 6). The individuals combined in the different samples (T10 vs. T12 and T14) also grow in different habitats and possess morphological differences [59], which would support the existence of a new entity (represented by pansies in T12 and T14) in addition to the typical *V. arvensis* type (sample T10). This latter would include forms that are directly linked to the ‘original’ more natural habitats of pansies (primary grasslands, wood margins, and clearings), are now settled in more recent synanthropic habitats (arable lands, resting fields, roadsides, and other altered habitats) and boosted by agricultural practices. The new type, generally overlooked due to confusion with other taxa (i.e., *V. kitaibeliana*; [59]), would have instead adapted to dry and open grasslands.

### 3.4. Inferences on Sect. Melanium Genome Evolution

Sect. *Melanium* is allotetraploid by origin and much of its diversification has occurred by further allopolyploidization up to at least 20*x* or 24*x* [1]. Within the *V. tricolor* group gene homoeolog numbers [1], *ITS* copy numbers [36], and genome size measurements [76] indicate that *V. kitaibeliana* (2*n* = 16) is 8*x*, *V. tricolor* (2*n* = 26) is 12*x*, and *V. arvensis* (2*n* = 34) is 16*x*; *V. hymettia* was not studied but having the same chromosome number as *V. kitaibeliana* it is probably 8*x* like that species.

The N-Net splits graph between species (Figure 3a) indicates a tree-like phylogenetic relationship between *V. tricolor*, *V. eugeniae*, and *V.* cf. *aetolica* on the one side and *V. arvensis*, *V. hymettia*, and *V. kitaibeliana* on the other. This indicates that among these species, subgenomes (i.e., as *5S-IGS* clusters) are either shared by all six species, shared by the three first ones or three last ones, or private to single species. In contrast, the high-polyploid *V. etrusca* (2*n* = 40) forms a split involving *V. tricolor* and *V. eugeniae*, which suggests that it may be an allopolyploid that possesses at least one subgenome with each of these two species. This finding is in agreement with the presumed reticulate polyploid relationships within sect. *Melanium* [1]. Taking chromosome counts and the distance plot at face value would suggest that the 16-ploid *V. arvensis* (2*n* = 34) originated by polyploidization from the common ancestor of the two octoploids *V. hymettia* and *V. kitaibeliana* (both 2*n* = 16); it might have been an autopolyploid or an allopolyploid involving very closely related parents.

In agreement with high ploidy in these taxa, the major *5S-IGS* sequence clusters and the multiple sub-clusters obtained with the phylogenetic analyses (Figure 4 and Figure 5) subtend the occurrence of different *5S* ribosomal lineages in all investigated species. This pattern can be explained with both intra-locus and intra-genomic (multiple loci on different chromosomes) variation [54], but the number of identified sequence lineages may not directly reflect the number of *5S* rDNA loci or chromosome numbers (and the ploidy level) of the investigated species owing to gene-level processes such as homogenization by concerted evolution and genome-level processes such as those following auto- or allopolyploidization, hybridization, and introgression [53]. However, the three to four *5S-IGS* sequence clusters retrieved in *V. tricolor* (Figure 4 and Figure 5) find a perfect match with the three/four *5S* rDNA loci identified in different populations of this species with FISH [55]. Following this argumentation, we might suggest an overall probable occurrence of two *5S* ribosomal loci in *V. kitaibeliana* and *V. hymettia*, (at least) three in *V.* cf. *aetolica* and *V. etrusca,* and four in *V. eugeniae*, with *V. arvensis* (two and three *5S* loci) comprising a variable number of cytotypes, similarly to *V. tricolor*. These suggested numbers of *5S* loci can hardly be reconciled with the chromosome numbers and the presumed ploidy levels of the investigated species. Indeed, increasing number (and diversity) of the *5S* lineages from *V. kitaibeliana* and *V. hymettia* (2*n* = 16) to *V. arvensis* (2*n* = 34) and *V. tricolor* (2*n* = 26) is consistent with different and reiterated allopolyploidization events and subsequent genome restructuring with partial loss of *5S* loci bearing chromosomes. It is interesting to note that similar dynamic and complex changes in rDNA genomic organization during speciation processes have been inferred in other plant systems as well, highlighting a general decoupling of the *5S* rDNA loci number with polyploidization events and chromosome numbers, a fact that could be explained by locus loss or homogenization of *5S* rDNA in allopolyploids over longer evolutionary times [51,77]. Therefore, the rate to which dysploidy affects the *5S* loci bearing chromosomes in sect. *Melanium* still remains an open question. Some nuclear gene markers have only opened the way to the identification of complex genome structures in *Viola*. In this view, *V. tricolor* has shown to be an excellent model to address cytotype characterization and evolution with *5S-IGS* and FISH. In future studies, traditional (banding) and advanced (FISH) data, coupled with extensive sequencing of the rDNA units and wider genomic sequencing approaches can surely contribute to a better definition of genome evolution in pansies.

Apart from the recent allopolyploidizations that have occurred within sect. *Melanium*, no whole-genome duplications (WGDs) have been detected in the history of this lineage since the ARTHγ event ca. 137 Ma in the common ancestor of Gunnerales and the Rosids [78]. It therefore seems reasonable to assume that the two major *5S-IGS* clades (Figure 6) that comprise 95% of the total *5S-IGS* sequences represent the two homoeologous subgenomes (CHAM and MELVIO) that were brought together by allotetraploidization c. 18 Ma ago in the ancestor of sect. *Melanium* [1] and that their subcluster structure contains the signals of the more recent allopolyploidizations during the diversification of the section. However, without the reference from a proper outgroup, this hypothesis cannot be confirmed. Ideal outgroup candidates would be those violets for which the occurrence of the CHAM and MELVIO subgenomes has been unambiguously assessed, such as tetraploids (e.g., *V.* (sect. *Melanium*) *dirimliensis* Blaxland or *V*. (sect. *Viola*) *odorata* L.) in combination with a diploid having the CHAM copy only (e.g., *V.* (sect. *Chamaemelanium*) *biflora* L.) and another diploid that is sister to both CHAM and MELVIO. It is however interesting to note that the two major clades are uneven in size, with the larger comprising ca. 75% of the *5S-IGS* sequences. Hence, *V. tricolor* and *V. arvensis* (besides *V. eugeniae*, *V. etrusca,* and *V.* cf. *aetolica*) have retained copies in both major clades, whereas *V. kitaibeliana* and *V. hymettia* only in the larger (Figure 5 and Figure 6). The smaller clade also appears to lack one of the two main clusters clearly observable in the larger clade (3 and 4). Together, these results indicate ongoing diploidization by selective loss of one of the main *5S-IGS* homoeologs, i.e., either CHAM or MELVIO, a pattern that is reflected in the complete loss of CHAM homoeologs for *ITS* in sect. *Melanium* [45,79].

## 4. Materials and Methods

### 4.1. Plant Material and DNA Extraction

Fifty-three individuals from four annual species of *Viola* sect. *Melanium* belonging to the ‘*V. tricolor* species complex’ were collected in the wild or obtained from UTV and ZAGR herbaria (acronyms according to Thiers, continuously updated [80]); five individuals belonging to perennial species of *Viola* sect. *Melanium* (*V. aethnensis*, *V. etrusca*, *V. eugeniae*, *V. frusinatae*, and *V.* cf. *aetolica*), and one individual from sect. *Viola* (*V. reichenbachiana*) were included in the dataset and used as outgroups (Table 5; details and maps provided as Supplementary File S1).

The adopted taxonomic circumscription followed [38]. Species identification and revisions were performed using the primary literature [2,3,13,38] and a critical examination of both fresh and dried plants, including relevant material available online. Species-representative chromosome counts were collected from previous studies [35,81]; ecological data of the sampling sites were also collected in the field or harvested from the specimen’s label (when available). Collections from *Loci classici* were prioritized when available (*V. hymettia*: Mount Hymetto (Greece); obtained samples of *V. kitaibeliana* from Hungary (‘Pannonia’) were revised to *V. arvensis*). DNA extractions were performed with NucleoSpin Plant II (Macherey-Nagel) and quantified with a NanoDrop spectrophotometer (Thermo Fisher Scientific, Waltham, MA, USA).

### 4.2. Plastid DNA and Nuclear 5S-HTS Procedures

The previously largely used *trnL* intron, *trnD-T,* and *matK* plastid markers were discarded based on literature surveys (low variation). Three highly variable markers (*trnH-psbA*, *Rps19-trnH*, *trnD-trnY*) were chosen based on recent plastome sequences of *Viola* [56] and amplified with specific primer pairs (*Rps19-trnH*: AATTTGATTCTTCGTCGCCG, CGCGCATGGTGGATTCACAATCC; *trnH-psbA*: CGCGCATGGTGGATTCACAATCC, GTTATGCATGAACGTAATGCTC; *trnD-trnY*: GGGTATCTCTTGAAATCCGGAAT, GGTTCAGGACATCTCTCTTTCAA). Thermocycling conditions were 94 °C for 3 min, followed by 35 cycles of 94 °C for 30 s, 55 °C (*trnH-psbA* and *Rps19-trnH*) or 58 °C (*trnD-trnY*) for 40 s, and 72 °C for 45 s (*trnH-psbA* and *Rps19-trnH*) or 1 min (*trnD-trnY*), with a final extension step of 10 min at 72 °C. PCR products were cleaned with GFX Illustra DNA Purification Kit (GE Healthcare); standardized aliquots were sent to Macrogen (http://www.macrogen.com) for sequencing, using the amplification primers. Fifty-eight individuals were analyzed. Sequences were deposited in GenBank under accession nos. OU782158-OU782215 (*rps*19-*trn*H), OU782216-OU782273 (*trn*H-*psb*A), and OU782421-OU782478 (*trn*D-*trn*Y).

For the HTS procedure, we prepared 19 DNA samples (20 ng) consisting of single and pooled individuals of the same species (Table 3; Supplementary File S1). The nuclear ribosomal *5S* intergenic spacer (*5S-IGS*) was amplified with the plant-specific primer pair CGTGTTTGGGCGAGAGTAGT and CTGCGGAGTTCTGATGG [53]. Paired-end Illumina sequencing (2 × 300 bp) was performed by LGC Genomics GmbH. Raw sequences were deposited in the Sequence Read Archive under BioProject PRJNA777417.

### 4.3. Bioinformatics and Statistical Tools

Obtained plastid DNA electropherograms were edited and checked visually with CHROMAS 2.6.5 (www.technelysium.com.au). Optimal multiple alignments, pairwise genetic distances, and the mean estimates of inter- and intragroup molecular divergence (for the concatenated markers) were performed with MEGA 7.014 [82]; DNASP 6.12.01 [83] was used to compute haplotype diversity parameters. A Median joining network of the combined plastid regions was run with Network 4.6.1.1 (http://www.fluxus-engineering.com/), treating gaps as 5th state and using the MJ algorithm with default parameters (equal weight of transversion/transition).

Pre-processing analysis of total Illumina paired-end reads (raw sequence data with adapters and primers clipped) was conducted with MOTHUR v.1.33.0 [84]. Contigs between read pairs were assembled using the ΔQ parameter [85]; reads were then dereplicated and chimeras were filtered using UCHIME in de novo mode [86]. The resulting unique (non-redundant) sequences (*5S-IGS* variants) with abundance ≥5 were aligned using MAFFT v.7 [87], manually checked using MEGA 7.014 [82], and used as total dataset for the subsequent statistical analyses. Phylogenetic analyses (trees and networks) were performed on a sub-dataset of unique *5S-IGS* sequences including all variants with abundance ≥25, excluding ca. 8% of shortest (<280 bp) and poorly alignable sequences.

Sequence structural features (length and GC content) were calculated with JEMBOSS 1.5 [88] and plotted with GGPLOT2 R package [89]. A preliminary identification of the obtained sequences was conducted based on sequence occurrence and its relative abundance in each sample. *5S-IGS* sequence variants resulting (near-) exclusive (>99.95%) o one taxon/sample were included in ‘specific’ classes, whereas those shared among taxonomically different samples were defined ‘ambiguous’. Graphical visualisation (pie charts) of the *5S-IGS* variants for each sample and relative different class assignation were performed by R [90]. Pairwise genetic distances and the mean estimates of inter- and intragroup molecular divergence (for *5S-IGS* variants) were built with MEGA 7.014 [82].

All maximum likelihood (ML) analyses were conducted with RAXML v.8.2.11 [91]. GTR + CAT (using the ‘extended majority-rule consensus’ criterion as bootstopping option, with up to 1000 bootstrap (BS) pseudoreplicates; [92]) and the GTR + gamma (with a non-parametric branch support of 1000 pseudoreplicates) were used as a models of nucleotide substitution to infer trees. Outputs were imported in iTOL (www.itol.embl.de); [93] for trees’ visualization and labeling. Unclear phylogenetic signals and general diversification patterns were explored with a planar (equal angle, parameters set to default) phylogenetic network generated with the Neighbor-Net (NNet) algorithm implemented in SPLITSTREE4 [94,95], using simple (Hamming) uncorrected pairwise (p-) distances.

Finally, in order to maximize the phylogenetic signal contained by such a short marker, we additionally coded the indels in the nucleotide alignment using the Simple Indel Coding [96] method implemented in SeqState [97] and analyzed both data partitions (nucleotides, indels) jointly using a method that sets a clock prior on branch lengths, i.e., a relative dating analysis, using BEAST v1.10.4 [98]. The nucleotide partition (528 characters) and coded indel partition (235 characters) were analyzed under a common lognormal relaxed clock set to 1 and a common Yule tree prior. Nucleotides were analyzed under a GTR + gamma model with 4 gamma rate categories. Coded indels were analyzed under a 1-rate + gamma model with 4 gamma rate categories. Two chains were run for a total of 130 million generations and sampled every 100,000 generations. To save computation time, a starting tree from exploratory analyses was used. Runs were monitored in Tracer v1.7.1 [99] to ensure all parameters reached an adequate effective sample size (ESS > 200). Runs were combined in LogCombiner (part of the BEAST package) after removal of a burn-in of 2 million generations each. The maximum credibility tree was summarized using TreeAnnotator (part of the BEAST package) and visualized in iTOL.

## 5. Conclusions

In this work, *V. arvensis* and *V. tricolor* showed plastid and nuclear genetic signatures pointing to a reticulated common origin. However, these two species are genetically different and progressively diverging, differentiating into multiple lineages likely driven by isolation, adaptation to local (human-triggered) habitats, hybridization, and introgression. Future assessment of the intra-specific diversity existing within these two species should be further inspected, as our data do not exclude the recognition of further (subspecific) taxonomic units. Ideally, investigations should adequately address cytology, eco-morphology, and nuclear genetic diversity in sympatric populations and related species across their distribution range.

*Viola kitaibeliana* and *V. hymettia* are morphologically hardly diagnosable (few significant micro-morphological traits coupled with a markedly variable macro-morphology [35,58]), possess identical chromosome numbers, a high but non-structured plastid diversity, and a similar low nuclear genetic diversity. Therefore, their identity and taxonomic autonomy cannot be considered as fully resolved, at least not on the Italian territory. What might be the most appropriate taxonomic status for them is yet to be defined; future studies should consider the Balkan Peninsula and west-central Europe as focal points of diversity that emerged in our study. With the current sampling of taxa, *V. arvensis* (2*n* = 34) appears to be an autopolyploid (or nearly so) derivate of the group consisting of *V. kitaibeliana* and *V. hymettia* (2*n* = 16).

Finally, the molecular approach applied in this work proved as a rapid procedure to inspect molecular differentiation within the investigated pansies, with a potential utility as a fast identification method of rare isolated lineages, hybridization, and introgression events, or the overall geno-taxonomic richness of environmental bulk samples. Nevertheless, the deep complexity of the genomic structure(s) in *Viola* sect. *Melanium* prevented reliable correlations between the *5S-IGS* lineages identified and genome organization.

Accumulating genomic data provide clear evidence that reticulation is a widespread phenomenon in plants [100] and the genomes of numerous modern angiosperms were molded by complex chromosome fractionation and rearrangement processes [101]. Sect. *Melanium* is an outstanding example, as preliminary essayed in this work on four annual pansies. Future studies will indeed profit from adequately designed samplings, including more perennial taxonomically well-circumscribed species and proper outgroups outside of the allopolyploid *Melanium* tangle, to understand whether the *5S-IGS* diversity may reflect the homoeology of CHAM, MELVIO, or both.

## Figures and Tables

**Table 1:** Estimates of mean evolutionary divergence over sequence pairs within and between taxa for the three combined plastid markers.

Taxon	N	Intra-	Inter-Group										H_tot	H_sh	Hd
*V. arvensis*	21	0.004		0.001	0.001	0.001	0.001	0.002	0.005	0.002	0.002	0.001	12	4	0.904
*V.* cf. *aetolica*	1	-	0.005		0.002	0.002	0.001	0.002	0.005	0.001	0.002	0.001	1	-	-
*V. hymettia*	8	0.001	0.003	0.006		0	0.002	0.002	0.005	0.002	0.002	0.002	6	2	0.893
*V. kitaibeliana*	7	0.001	0.003	0.006	0.001		0.002	0.002	0.005	0.002	0.002	0.002	5	3	0.857
*V. tricolor*	16	0.002	0.005	0.004	0.007	0.007		0.002	0.005	0.002	0.002	0.001	8	1	0.7
*V. frusinatae*	1	-	0.007	0.005	0.008	0.007	0.006		0.005	0.002	0.002	0.002	1	-	-
*V. reichenbachiana*	1	-	0.048	0.045	0.047	0.047	0.05	0.049		0.005	0.005	0.005	1	-	-
*V. etrusca*	1	-	0.006	0.003	0.006	0.006	0.005	0.006	0.048		0.002	0.001	1	-	-
*V. eugeniae*	1	-	0.009	0.006	0.01	0.009	0.007	0.007	0.05	0.006		0.002	1	-	-
*V. aethnensis*	1	-	0.004	0.001	0.005	0.005	0.003	0.005	0.046	0.001	0.006		1	-	-

Standard error estimates are shown above the diagonal; color graduation—minimum (crimson) to maximum (dark green) values. N—Number of sequences; H_tot—total haplotypes found in each taxon; H_sh—number of shared haplotypes; Hd—haplotype diversity.

**Table 2:** Estimates of mean evolutionary divergence over sequence pairs for the nuclear *5S*-HTS sequences with abundance ≥5.

	Sample	d	S.E.	Sample	d	S.E.	Sample	d	S.E.	Sample	d	S.E.
*V. arvensis*	T14	0.060	0.010	-	-	-	-	-	-	-	-	-
*V. cfr. aetolica*	T16	0.100	0.010	-	-	-	-	-	-	-	-	-
*V. kitaibeliana*	T17	0.060	0.010	T18	0.070	0.010	T20	0.070	0.010	-	-	-
*V. tricolor*	T2	0.120	0.010	T6	0.120	0.010	T8	0.110	0.010	T9	0.140	0.010
*V. hymettia*	T24	0.070	0.010	T26	0.060	0.010	T28	0.060	0.010	-	-	-
*V. etrusca*	T29	0.130	0.010	-	-	-	-	-	-	-	-	-
*V. eugeniae*	T30	0.130	0.010	-	-	-	-	-	-	-	-	-

color graduation: minimum (crimson) to maximum (dark green) values.

**Table 3:** Intragenomic mean divergence.

		Intra-	Inter-Species						
	N		*V.* cf. *aetolica*	*V. arvensis*	*V. etrusca*	*V. eugeniae*	*V. hymettia*	*V. kitaibeliana*	*V. tricolor*
*V.* cfr. *aetolica*	1	-							
*V. arvensis*	7	0.071	0.117						
*V. etrusca*	1	-	0.114	0.118					
*V. eugeniae*	1	-	0.123	0.118	0.124				
*V. hymettia*	8	0.067	0.12	0.073	0.119	0.119			
*V. kitaibeliana*	5	0.071	0.119	0.073	0.118	0.118	0.07		
*V. tricolor*	4	0.118	0.122	0.11	0.124	0.128	0.11	0.111	

d—distance; S.E.—Standard error. (b) intra- and interspecific mean divergence; N—number of individuals; color graduation: minimum (crimson) to maximum (dark green) values.

**Table 4:** Mean divergence among the ‘V. tricolor species complex’ samples.

	N	T10	T12	T14	T17	T18	T2	T20	T21	T24	T25	T26	T27	T28	T6	T8	T9
*V. arvensis*-T10	4																
*V. arvensis-*T12	2	0.075															
*V. arvensis*-T14	1	0.073	0.065														
*V. kitaibeliana*-T17	1	0.068	0.066	0.063													
*V. kitaibeliana*-T18	1	0.075	0.072	0.070	0.064												
*V. tricolor*-T2	1	0.109	0.111	0.111	0.107	0.113											
*V. kitaibelian*a-T20	1	0.080	0.074	0.073	0.067	0.073	0.111										
*V. kitaibeliana*-T21	2	0.078	0.072	0.072	0.069	0.073	0.112	0.076									
*V. hymettia*-T24	1	0.072	0.069	0.067	0.059	0.067	0.108	0.069	0.071								
*V. hymettia*-T25	2	0.066	0.067	0.063	0.055	0.064	0.108	0.069	0.069	0.059							
*V. hymettia*-T26	1	0.074	0.072	0.069	0.061	0.070	0.108	0.070	0.075	0.064	0.060						
*V. hymettia*-T27	3	0.067	0.068	0.065	0.058	0.065	0.108	0.070	0.069	0.061	0.055	0.062					
*V. hymettia*-T28	1	0.081	0.074	0.074	0.069	0.074	0.112	0.073	0.075	0.071	0.071	0.073	0.071				
*V. tricolor*-T6	1	0.105	0.107	0.107	0.105	0.110	0.111	0.111	0.108	0.106	0.106	0.109	0.107	0.111			
*V. tricolor*-T8	1	0.098	0.096	0.096	0.094	0.098	0.110	0.098	0.097	0.095	0.096	0.098	0.096	0.098	0.105		
*V. tricolor*-T9	1	0.116	0.112	0.114	0.111	0.115	0.125	0.113	0.112	0.112	0.115	0.116	0.115	0.112	0.122	0.116	

N—number of individuals in each sample; color graduation: minimum (crimson) to maximum (dark green) values.

**Table 5:** Investigated dataset.

Id	Origin	Sampling Site	Taxon	Performed Analysis	
				Plastid DNA	Nuclear rDNA
Vt-1	Switzerland	Novaggio, Lugano	*Viola tricolor* L. subsp*. subalpina* Gaudin	*	
Vt-2	Germany	Kreis Bad Neustadt/Saale, Bayern	*V. tricolor* L. subsp. *tricolor*	*	*
Vt-3	Italy	Casalvieri, Frosinone	*Viola tricolor* L. subsp. *tricolor*	*	
Va-1	Italy	Aquilonia, Avellino	*Viola arvensis* Murray	*	*
Va-2	Italy	Roaschia, Cuneo	*Viola arvensis* Murray	*	
Vk-1	Italy	Mt Navegna, Rieti	*Viola kitaibeliana* Schultes	*	*
Vk-2	Italy	Bassano Scalo, Orte, Viterbo	*Viola kitaibeliana* Schultes	*	*
Vh-1	Italy	Le Vigne, Ofena, L’Aquila	*Viola hymettia* Boiss. & Heldr.	*	*
Vh-2	Italy	Mt Palanzana, Viterbo	*Viola hymettia* Boiss. & Heldr.	*	*
Vh-3	Italy	Marchionato, Vetralla, Viterbo	*Viola hymettia* Boiss. & Heldr.	*	*
Va-3	Italy	Acque Albule, Tivoli, Roma	*Viola arvensis* Murray	*	*
Va-4	Italy	Bagnaccio, Viterbo	*Viola arvensis* Murray	*	*
Va-6	Italy	Basovizza, Trieste	*Viola arvensis* Murray	*	
Vh-4	Italy	Vernole, Acquarica, Lecce	*Viola hymettia* Boiss. & Heldr.	*	*
Va-7	Italy	Stacchini, Tivoli, Roma	*Viola arvensis* Murray	*	*
Va-25	France	Plateau de Guicule, Le Lavandou	*Viola arvensis* Murray	*	
Vh-5	Italy	Rugoro Grosso, Biancavilla, Catania	*Viola hymettia* Boiss. & Heldr.	*	*
Vh-6	Italy	Cannarozzo, Piazza Armerina, Enna	*Viola hymettia* Boiss. & Heldr.	*	*
Vh-7	Italy	Pineta di Biancavilla, Catania	*Viola hymettia* Boiss. & Heldr.	*	*
Vh-8	Greece	Mt Imittos, Attiki	*Viola hymettia* Boiss. & Heldr.	*	*
Vk-5	Greece	Mt Pendelikon, Attiki	*Viola kitaibeliana* Schultes	*	*
Vk-6	France	Clermont-L’Hérault	*Viola kitaibeliana* Schultes	*	
Vk-7	Spain	La Palomera, Córdoba	*Viola kitaibeliana* Schultes	*	*
Vk-8	Spain	Los Villares/CO-3408, Córdoba	*Viola kitaibeliana* Schultes		*
Vk-9	Croatia	Šušanj Cesarički, Velebit	*Viola kitaibeliana* Schultes	*	
Va-26	Croatia	Radoboj, Hrvatsko Zagorje	*Viola arvensis* Murray	*	
Va-8	Italy	Barrea, l’Aquila	*Viola arvensis* Murray	*	*
Va-9	Italy	San Gregorio da Sassola, Roma	*Viola arvensis* Murray	*	*
Va-10	Italy	Mt Cetona, Siena	*Viola arvensis* Murray	*	*
Va-11	Croatia	Zapadni kolodvor, Zagreb	*Viola arvensis* Murray	*	
Va-12	Croatia	Kozjak, Malačka, Dalmacija	*Viola arvensis* Murray	*	
Vt-4	Italy	Firenzuola, Firenze	*Viola tricolor* L. subsp. *tricolor*	*	
Vt-5	Czec Republic	Ketkovice, Brno-Venkov, Moravia	*Viola tricolor* L. subsp*. subalpina* Gaudin	*	
Vt-6	Czec Republic	Pavlov, Břeclav, Moravia	*Viola tricolor* L. subsp*. subalpina* Gaudin	*	
Va-16	Czec Republic	Pavlov, Břeclav, Moravia	*Viola arvensis* Murray	*	
Va-27	Czec Republic	Pavlov, Břeclav, Moravia	*Viola arvensis* Murray	*	
Va-28	Slovacia	Nitra-Zobor, Nitra	*Viola arvensis* Murray	*	
Va-18	Czec Republic	Ráby, distr. Pardubice, Bohemia	*Viola arvensis* Murray	*	
Vt-8	Bosnia–Herzeg.	Planina Dinara, Badanj	*Viola tricolor* L. subsp. *subalpina* Gaudin	*	
Va-20	Croatia	Gornje Sitno, Mosor, Dalmacija	*Viola arvensis* Murray	*	
Va-21	France	Ludesse, Haute-Loire	*Viola arvensis* Murray	*	
Vt-9	France	Llo, Pyrénées-Orientales	*Viola tricolor* L.	*	*
Vt-10	France	Beuil, Alpes-Maritimes	*Viola tricolor* L. subsp*. subalpina* Gaudin	*	
Vt-12	France	La Motte-en-Champsaur, Alpes-Maritimes	*Viola tricolor* L. subsp*. subalpina* Gaudin	*	
Va-23	France	Saint-Christophe-en-Oisans, Isère	*Viola arvensis* Murray	*	
Vt-13	Italy	Rionero in Vulture, Potenza	*Viola tricolor* L. subsp. *tricolor*	*	
Vt-14	Italy	Chiavari, Genova	*Viola tricolor* L. subsp. *tricolor*	*	
Vt-15	Italy	Vinadio, Cuneo	*Viola tricolor* L. subsp*. subalpina* Gaudin	*	
Vt-16	Italy	Cortina D’Ampezzo, Belluno	*Viola tricolor* L. subsp. *subalpina* Gaudin	*	
Vt-17	Bulgaria	Beli Iskar, Rila Mountain	*Viola tricolor* L.	*	*
Vt-18	Poland	Płociczno, distr. Gmina Bakałarzewo	*Viola tricolor* L. subsp. *tricolor*	*	*
Va-24	Poland	Nadleśnictwo Kędzierzyn	*Viola arvensis* Murray	*	
Vk-13	Italy	Palazzolo, Vasanello, Viterbo	*Viola arvensis* Murray	*	
Ve	Italy	Poggio Mutti, Montieri, Grosseto	*Viola etrusca* Erben	*	*
Vg	Italy	Selva Rotonda, Cittareale, Rieti	*Viola eugeniae* Parl. subsp. *eugeniae*	*	*
Vn	Italy	Accadia, Foggia	*Viola aethnensis* (DC.) Strobl.	*	
Vf	Italy	Casalattico, Frosinone	*Viola frusinate* Ricceri & Moraldo	*	
Vae	Albania	Mat River, Klos	*Viola* cf. *aetolica*	*	*
Vr	Italy	Castel Morrone, Caserta	*Viola reichenbachiana* Jord. ex Boreau	*	

* = analyzed samples.

## Data Availability

Used alignments, inference files, and additional descriptive files are available at: https://doi.org/10.6084/m9.figshare.17698157.v2. All other relevant data are contained within the manuscript and its supporting materials.

## Supplementary Materials

The following are available online at https://www.mdpi.com/article/10.3390/plants11101294/s1, Supplementary File S1 [Excel]: Description of the investigated dataset, including the sampling design, voucher ID, sampling sites with geographical and ecological details, collector, sampling date, original/revised name, and geographical maps with the distribution of the samples used for the plastid and the nuclear 5S-HTS DNA analysis. Supplementary File S2 (Excel): description of the worked 5S-HTS dataset, including pre-processing steps and details on the distribution and main structural features of the HTS sequence reads in the 19 investigated samples.
